# Uric Acid Impairs Insulin Signaling by Promoting Enpp1 Binding to Insulin Receptor in Human Umbilical Vein Endothelial Cells

**DOI:** 10.3389/fendo.2018.00098

**Published:** 2018-03-26

**Authors:** Eliezer J. Tassone, Antonio Cimellaro, Maria Perticone, Marta L. Hribal, Angela Sciacqua, Francesco Andreozzi, Giorgio Sesti, Francesco Perticone

**Affiliations:** ^1^Department of Medical and Surgical Sciences, Magna Græcia University, Catanzaro, Italy; ^2^Department of Experimental and Clinical Medicine, Magna Græcia University, Catanzaro, Italy

**Keywords:** uric acid, insulin resistance, insulin signaling, ectonucleotide pyrophosphatase phosphodiesterase, endothelium, nitric oxide, vascular damage

## Abstract

High levels of uric acid (UA) are associated with type-2 diabetes and cardiovascular disease. Recent pieces of evidence attributed to UA a causative role in the appearance of diabetes and vascular damage. However, the molecular mechanisms by which UA induces these alterations have not been completely elucidated so far. Among the mechanisms underlying insulin resistance, it was reported the role of a transmembrane glycoprotein, named either ectonucleotide pyrophosphatase/phosphodiesterase 1 (ENPP1) or plasma cell antigen 1, which is able to inhibit the function of insulin receptor (I_R_) and it is overexpressed in insulin-resistant subjects. In keeping with this, we stimulated human umbilical vein endothelial cells (HUVECs) with insulin and UA to investigate the effects of UA on insulin signaling pathway, testing the hypothesis that UA can interfere with insulin signaling by the activation of ENPP1. Cultures of HUVECs were stimulated with insulin, UA and the urate transporter SLC22A12 (URAT1) inhibitor probenecid. Akt and endothelial nitric oxide synthase (eNOS) phosphorylation levels were investigated by immunoblotting. ENPP1 binding to I_R_ and its tyrosine phosphorylation levels were tested by immunoprecipitation and immunoblotting. UA inhibited insulin-induced Akt/eNOS axis. Moreover, UA induced ENPP1 binding to I_R_ that resulted in an impairment of insulin signaling cascade. Probenecid reverted UA effects, suggesting that UA intracellular uptake is required for its action. In endothelial cells, UA directly interferes with insulin signaling pathway at receptor level, through ENPP1 recruitment. This evidence suggests a new molecular model of UA-induced insulin resistance and vascular damage.

## Introduction

Uric acid (UA) is the end-product of purine metabolism, with both anti-oxidant and pro-oxidant properties; it has been demonstrated to have a key role in the redox process related to oxidative stress (1) that is involved in vascular damage and metabolic alterations (2–4). In addition, other pieces of evidence show that high levels of UA are able to predict myocardial ischemia and cerebrovascular events in general population (5, 6) and in post-menopausal women (7), and then mortality in patients affected by heart failure (8) and coronary heart disease (9). Furthermore, data from Brisighella Study documented that UA levels are associated with abnormal cardiac performance and impaired cognitive function already in a preclinical stage (10, 11).

In keeping with this, we previously demonstrated that serum UA levels, independently from classical cardiovascular risk factors, are associated with endothelial dysfunction (12) and the appearance of overt diabetes (13) in untreated hypertensive patients. In particular, we demonstrated that the increase of 1 mg/dl of serum UA levels reduces of 40% endothelium-dependent vasodilation. This phenomenon is due to a reduced bioavailability of nitric oxide (NO), secondary to an excess of reactive oxygen species (2, 14). In addition, recent pieces of evidence suggest that UA alone or in association with endothelial dysfunction plays a causative role in the appearance of incident diabetes (13, 15–17). This association may be justified by the pro-oxidant and pro-inflammatory actions of UA that affect both glucose homeostasis and insulin sensitivity, promoting clinically evident diabetes (18–20). Interestingly, we also demonstrated that UA levels are associated with an impairment of glucose tolerance status during an oral glucose tolerance test in hypertensive subjects (21).

However, despite the well-known correlation existing between UA and the risk of diabetes, the molecular processes by which UA can induce insulin resistance are not completely clear. In particular, it is still uncertain whether UA can directly inhibit the insulin signaling pathway and whether it has a causal role in determining insulin resistance beyond the oxidative stress. Insulin resistance recognizes multiple abnormalities that may occur in the insulin signaling pathway. Some of these alterations involve the insulin receptor (I_R_), whereas others may impair one or more of the downstream signaling steps (22). The plasma membrane enzyme, named ectonucleotide pyrophosphatase/phosphodiesterase 1 (ENPP1), also known as plasma cell antigen-1, has been shown to inhibit I_R_ function and having high expression levels in cells of insulin-resistant subjects (23). ENPP1 is a transmembrane glycoprotein that regulates nucleotide metabolism (24), it is located in the plasma membrane and in the endoplasmic reticulum and expressed in all major insulin target tissues. ENPP1 is a homodimer with an enzymatic activity cleaving sugar-phosphate, phosphosulfate, pyrophosphate, and phosphodiesterase. There are pieces of evidence that ENPP1, binding to α-subunit of I_R_ (I_R_α), impairs I_R_ signaling by inhibiting its autophosphorylation, and consequently insulin receptor substrate-1 phosphorylation and glucose transport (25) contributing, thus, to the development of insulin resistance (26).

On the basis of these observations, we designed this study to evaluate the molecular effects of UA on insulin signaling pathway in human umbilical vein endothelial cells (HUVECs). In particular, we tested the hypothesis that UA can interfere with insulin signaling throughout the activation of ENPP1.

## Materials and Methods

### Cell Culture

Human umbilical vein endothelial cells were obtained from ScienCell Research Laboratories (San Diego, CA, USA) and maintained in endothelial cell medium according to the manufacturer’s instructions. Growth medium was replaced every 48/72 h and cells were grown until 95–100% confluent. We used the same batch of HUVECs from a single donor for all the experiments, at different passages ranging from 2 to 6.

### Insulin Signaling Experiments

Human umbilical vein endothelial cells, seeded in 100 or 150 mm tissue culture dishes, were cultured until 80% confluent. UA (Ultrapure, Sigma-Aldrich, Milan, Italy) was dissolved in pre-warmed (37°C) medium; the mixture was then warmed again (37°C, 30 min) and sterile filtered. Probenecid, a urate transporter (SLC22A12, URAT1) inhibitor, was added from a concentrated stock, prepared in NaOH at the maximal allowed concentration (50 mg/dl), to 1 mM treatment concentration (27). HUVECs were maintained in starvation medium (without FBS and growth factor supplements, with 0.1% BSA) for 18 h and then incubated with UA, resuspended in fresh starvation medium, for 7 and 30 min. In the co-treatment experiments Probenecid was added for 30 min to UA supplemented medium. When indicated, HUVECs were stimulated with insulin (10^−7^M) for the last 7 min of UA or UA+Probenecid incubation.

### Western Blot Analysis and Immunoprecipitation Assays

Cells were lysed in buffer containing 1.5% NP-40 and analyzed by immunoprecipitation and Western blotting as previously described (28–31). Briefly, equal amounts of cell lysates were either incubated overnight with anti-I_R_α antibody or directly loaded to SDS-PAGE gels. For immunoprecipitation experiments, immune complexes were collected by incubation with protein A-Sepharose and resuspended in Laemmli buffer before loading to SDS-PAGE gels. Gels were then transferred to nitrocellulose membranes and immunoblotted with the appropriate primary antibodies, according to standard protocols. To evaluate ENPP1 binding to I_R_ and I_R_ tyrosine phosphorylation, the I_R_α immunoprecipitated proteins were immunoblotted, with an anti-ENPP1 and or an anti-tyrosine phosphorylated proteins antibody. To normalize for protein levels, the blots were stripped and reprobed with primary antibodies against the total unphosphorylated form of the appropriate protein. Primary antibodies used for this study were purchased from *Cell Signaling Technology*, Beverly, MA, USA (eNOS, phospho-eNOS Ser^1177^, Akt and phospho-Akt Ser^473^, anti-I_R_α, and anti-ENPP1) or from Merck Millipore (clone 4G10). Blots were visualized using appropriate peroxidase-conjugated secondary antibodies followed by enhanced chemiluminescence detection, and band densities were quantified by densitometry using an ImageJ software.

### Statistical Analysis

All results are given as mean fold variation ± SE over control. Statistical differences were assessed by Student’s *t*-test. Values of *P* < 0.05 were considered statistically significant. Analyses were performed with GraphPad Prism version 6 software.

## Results

### Effects of UA on Insulin-Stimulated Activation of Akt/Endothelial Nitric Oxide Synthase (eNOS) Signaling

As well-known, insulin binding to I_R_ is able to activate a dual signaling pathway: the PI3K/Akt signaling pathway, promoting metabolic effects, and the MAPK-related signaling pathway, promoting cellular proliferation, differentiation, and gene expression. In endothelial cells, the metabolic and hemodynamic effects of insulin are mediated by the activation of PI3K/Akt axis that induces a phosphorylation cascade leading to glycogen synthesis and glucose uptake and, above all, eNOS activation that increases NO production promoting in turn vasodilation and improving endothelial function.

**Figure 1:**
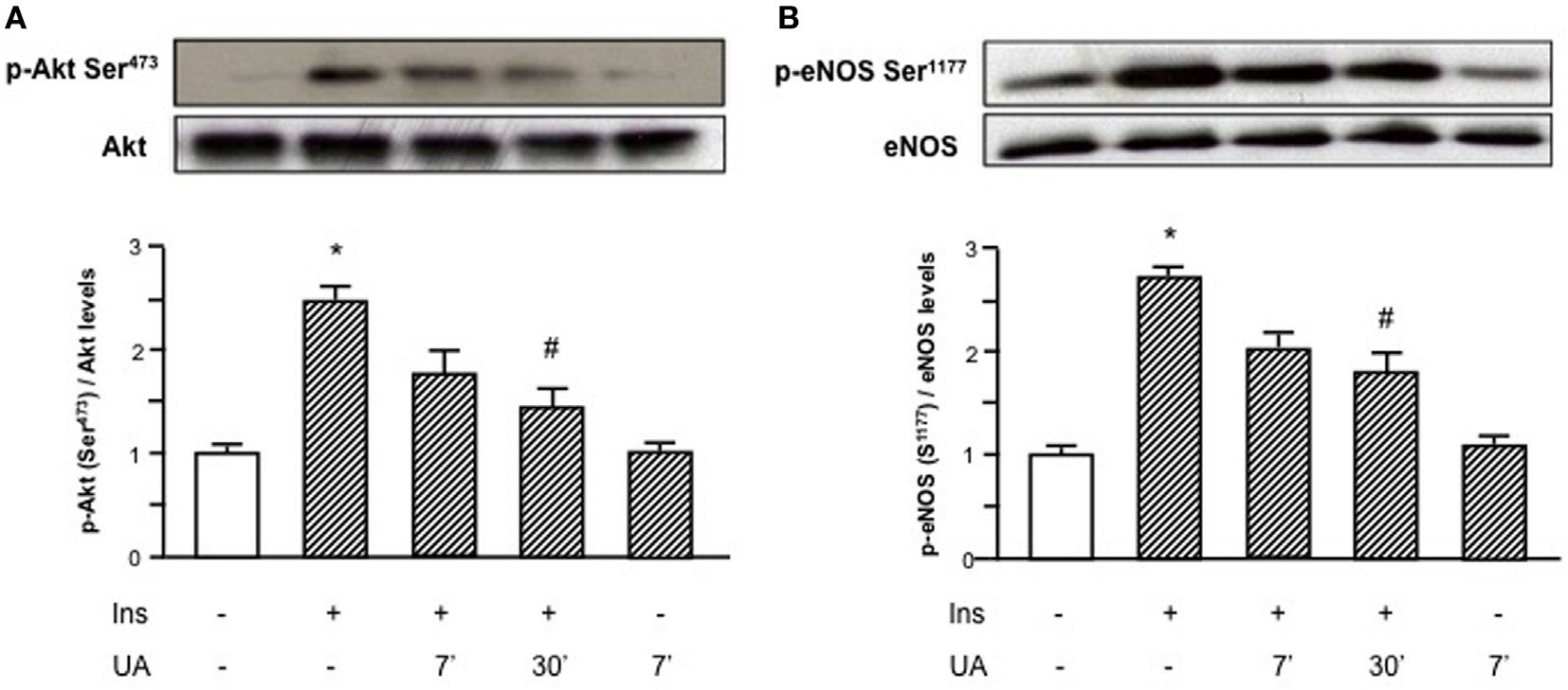
Effects of uric acid (UA) on insulin-induced Akt **(A)** and endothelial nitric oxide synthase (eNOS) **(B)** phosphorylation at Ser^473^ and Ser^1177^, respectively. Exposure of human umbilical vein endothelial cells to insulin induces a rapid activation of Akt/eNOS axis; in presence of UA this effect is largely reduced, particularly at 30′. To normalize the blots for protein levels, after being immunoblotted with anti-phosphospecific antibodies, the blots were stripped and reprobed with anti-Akt or anti-eNOS total forms. Bars represent means ± SE, expressed as relative change in comparison with the basal value, for three independent experiments and autoradiographs of a representative experiment are shown. *P < 0.05 vs basal value; ^#^*P* < 0.05 vs insulin.

As shown in Figure 1, the exposure of HUVECs to UA negatively affected the insulin downstream signaling. In particular, UA inhibited the insulin-induced serine phosphorylation of Akt (Ser^473^, Figure 1A) and eNOS (Ser^1177^, Figure 1B), with maximal effect at 30′ exposition.

### Effects of UA on ENPP1 Recruitment and Related Tyrosine Phosphorylation Inhibition of I_R_

**Figure 2:**
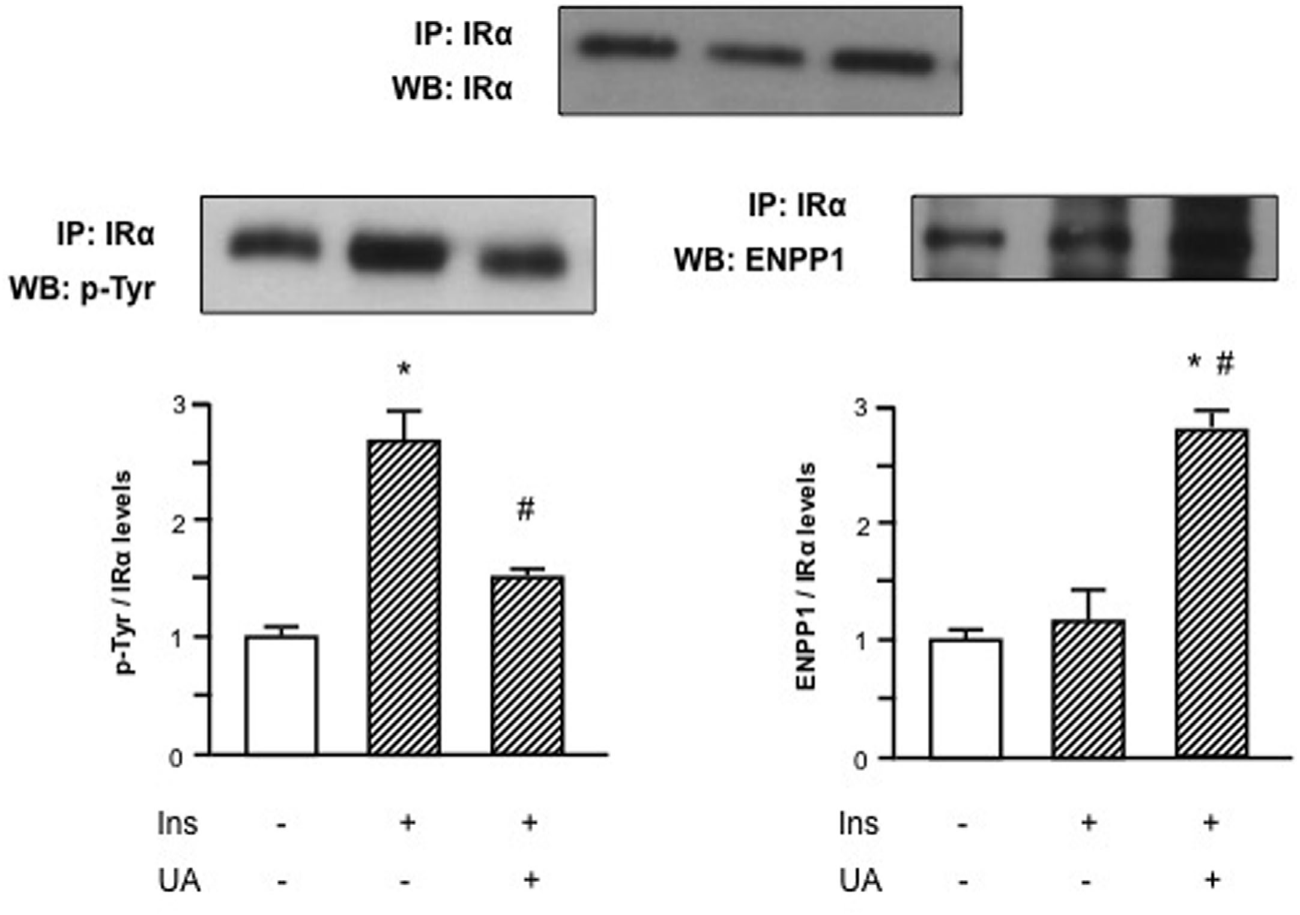
Effects of uric acid (UA) on ectonucleotide pyrophosphatase/phosphodiesterase 1 (ENPP1) binding to α-subunit of insulin receptor (I_R_α) and inhibition of tyrosine phosphorylation of I_R_ in human umbilical vein endothelial cells (HUVECs). Insulin-induced tyrosine phosphorylation of I_R_, as first step in the insulin signaling pathway, is prevented by the exposure of HUVECs to UA; this effect might be explained by the binding of ENPP1 to I_R_α induced by UA. To normalize the blots for protein levels, after being immunoblotted with anti-ENPP1 or anti-p-Tyr total antibodies, the blots were stripped and reprobed with anti-I_R_α total levels. Each bar represents the mean ± SD of three independent experiments and autoradiographs of a representative experiment are shown. *P < 0.05 vs basal value; ^#^*P* < 0.05 vs insulin.

Given the inhibitory effect of UA on Akt/eNOS signaling pathway, we hypothesized that UA could exert a direct action on insulin signaling at membrane level. Since preliminary results demonstrated that the maximal inhibitory effect of UA is observed at 30′ exposure, we used this time point for subsequent experiments. Initially, we performed an immunoprecipitation test to evaluate the molecular effect of UA on I_R_ tyrosine phosphorylation. As shown in Figure 2, UA was able to reduce insulin-induced I_R_ tyrosine phosphorylation.

We then speculated that this UA effect might be mediated by an increased binding of I_R_ to ENPP1, a well-known inhibitor of I_R_ auto-phosphorylation that has been shown to be overactive in condition of insulin resistance (24–27). In Figure 2, we can observe that UA induced ENPP1 binding to I_R_α in presence of insulin, suggesting a possible counter-regulatory mechanism.

### Effect of the Urate Transporter SLC22A12 (URAT1) Inhibitor Probenecid on UA-Mediated Effects on Insulin Signaling

To assess if the action of UA on insulin signaling required an intracellular uptake of UA, we treated HUVECs with Probenecid, a known organic anion transporter inhibitor. In fact, it has been demonstrated that Probenecid at 1 mM efficaciously inhibits SLC22A12 (URAT1)—mediated entry of UA in endothelial cells (28).

**Figure 3:**
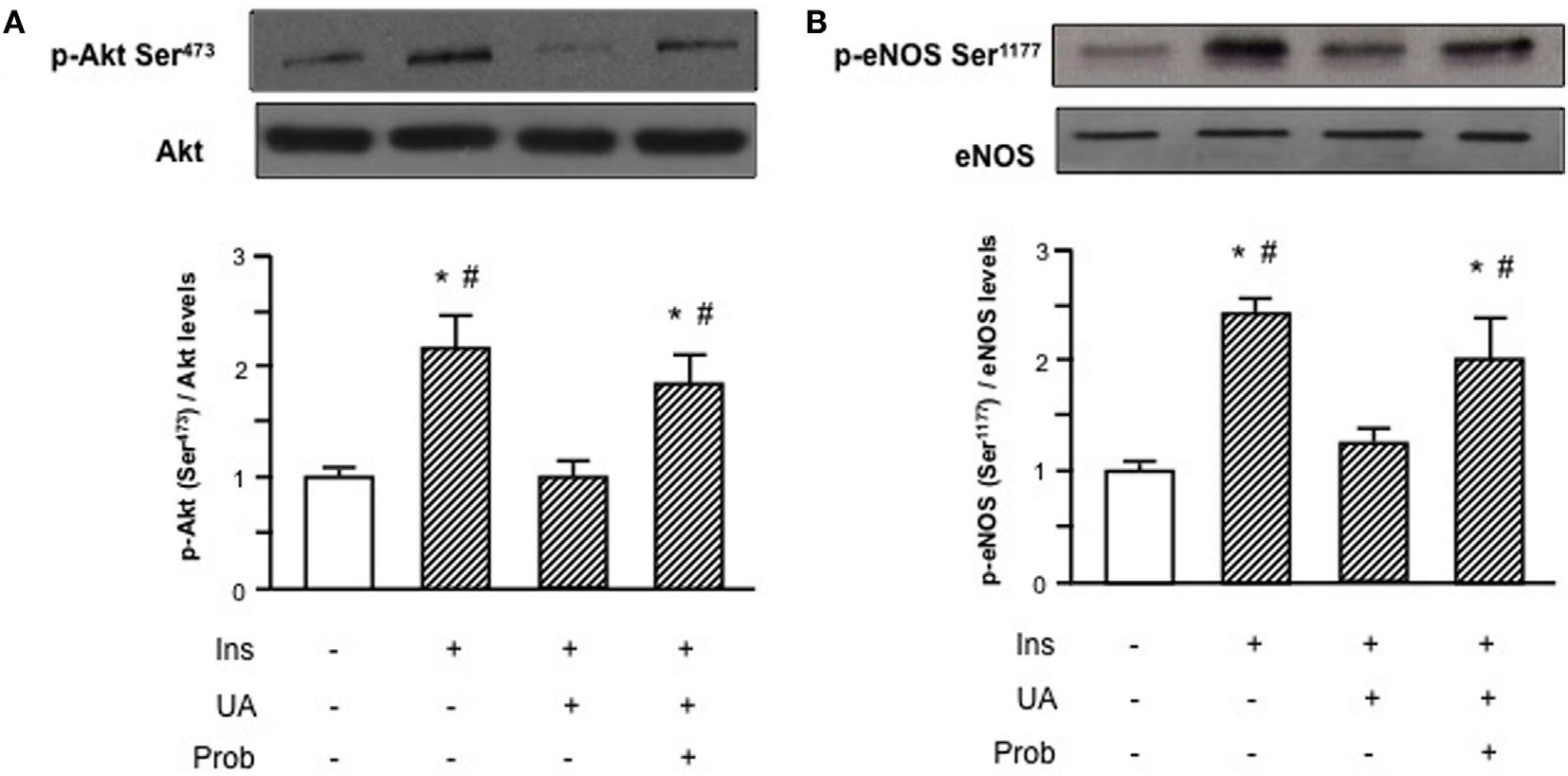
Effects of the SLC22A12 (URAT1) inhibitor Probenecid on uric acid (UA)-mediated effects on insulin downstream signaling in human umbilical vein endothelial cells (HUVECs). Exposure of HUVECs to Probenecid (1 mM, 30′) restores Akt **(A)** and endothelial nitric oxide synthase (eNOS) **(B)** insulin-induced phosphorylation levels in presence of UA. To normalize the blots for protein levels, after being immunoblotted with anti-phosphospecific antibodies, the blots were stripped and reprobed with anti-Akt or anti-eNOS total levels. Each bar represents the mean ± SD of three independent experiments and autoradiographs of a representative experiment are shown. *P < 0.05 vs basal value; ^#^*P* < 0.05 vs insulin+UA.

**Figure 4:**
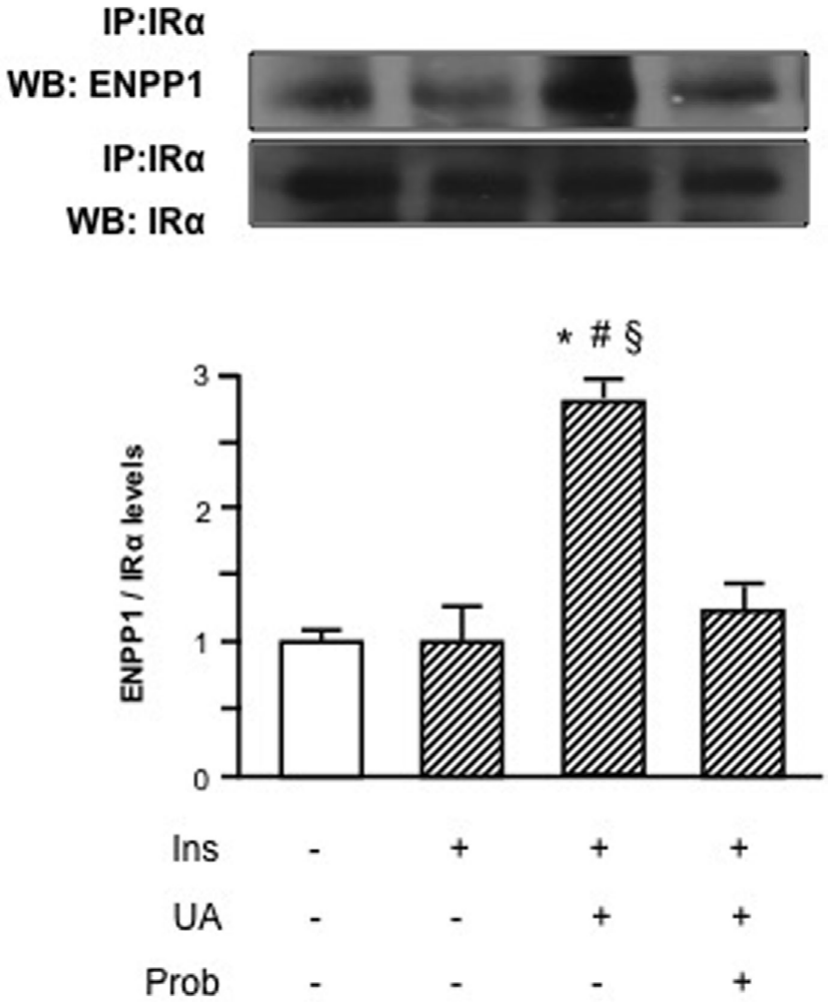
Effects of the SLC22A12 (URAT1) inhibitor Probenecid on uric acid (UA)-mediated ectonucleotide pyrophosphatase/phosphodiesterase 1 (ENPP1) binding to insulin receptor (I_R_) in human umbilical vein endothelial cells (HUVECs). In presence of insulin and UA, there is a significant reduction of I_R_ binding to ENPP1 when HUVECs are treated with Probenecid. To normalize the blots for protein levels, after being immunoblotted with anti-ENPP1, the blots were stripped and reprobed with anti-α-subunit of insulin receptor (I_R_α) total levels. Each bar represents the mean ± SD of three independent experiments and autoradiographs of a representative experiment are shown. *P < 0.05 vs basal value; ^#^*P* < 0.05 vs insulin; ^§^*P* < 0.05 vs insulin+UA+Probenecid.

HUVECs exposure to Probenecid restored Akt insulin-induced phosphorylation levels in presence of UA and the increased Akt phosphorylation resulted, in turn, in an enhanced eNOS activation (Figure 3). Given the effect on insulin downstream signaling, we addressed the question if Probenecid could interfere with UA-mediated recruitment of ENPP1 and related ENPP1/I_R_ association. As shown in Figure 4, Probenecid was able to re-establish basal association levels of ENPP1 to I_R_ in presence of insulin and UA.

## Discussion

In this study, we demonstrated for the first time that UA promotes the binding of ENPP1 to I_R_, inhibiting its activation. This evidence clearly shows that UA directly interferes with the insulin signaling pathway in a way totally independent of its ability to increase the oxidative stress and the inflammatory burden at cellular level. Thus, data obtained from this study demonstrate the hypothesis that UA has a primary role in the appearance and progression of insulin resistance and, then, in the development of diabetes. In fact, the demonstration that UA is directly able to impair the insulin signaling pathway, by inhibiting the cellular trigger of insulin signal at receptor level, attributes to hyperuricemia a causal independent role in the pathogenetic mechanisms of insulin resistance beyond its mediator role in the oxidative stress and inflammation.

Identification of hyperuricemia as a risk factor for diabetes has been uncertain for many decades because it has been considered a consequence of insulin resistance rather than a cause (32, 33). In the recent years, several studies have established that increased levels of UA are associated with insulin resistance (34), obesity (17), and new onset of type-2 diabetes (15–17, 35). UA levels are associated with oxidative stress (1, 14, 33) and mild-inflammation (18–20), which in turn contribute to the onset of diabetes (19, 36). Moreover, in a recent *in vivo* study it was seen that hyperuricemia caused by fructose plays a role in the pathogenesis of metabolic syndrome (37). Thus, our data contribute to explain all these findings suggesting that high serum UA precedes the development of overt type-2 diabetes.

Notably in the present study, we were also able to show that UA has a key role in reducing Akt–eNOS axis activity that is involved in the normal vascular function; it is clearly demonstrated that its impairment induces endothelial dysfunction that represents the first step in the atherosclerotic process (38). We previously reported a linear relationship between UA and endothelial dysfunction (12) and, subsequently, we also observed that both high sensitivity C-reactive protein concentrations and impaired endothelial function are independent predictors of new diabetes (19). Recently, we showed that hypertensive subjects have an increased risk to develop type 2 diabetes if they present both an impaired endothelium-dependent vasodilation and hyperuricemia; this increased risk is likely mediated by a condition of mild inflammation (13, 19). These findings agree with those of other studies demonstrating that oxidative stress caused by hyperuricemia has a role in the development of vascular damage. These pieces of evidence are reinforced by two potentially important biological actions of UA that lead to an impaired endothelium-dependent vasodilation: first, UA promotes mild-inflammation, as documented by increased CRP expression (38–40); second, it increases oxidative stress in several cell types, such as vascular smooth muscle cells and murine adipocytes, despite its antioxidant effect in an extracellular environment (41, 42).

Uric acid also stimulates vascular smooth muscle cells through a specific organic anion transport pathway, platelet-derived growth factor-dependent proliferation, monocyte chemoattractant protein-1 and cyclooxygenase-2-dependent thromboxane synthesis, and through the activation of renin–angiotensin system (43, 44), all factors that participate to the development and progression of atherosclerosis. Physiologically, endothelium regulates a number of biological processes implicated in vascular homeostasis, including the balance of pro-thrombotic and antithrombotic factors, platelet aggregation, leukocytes and monocytes adhesion, and vascular smooth muscle cells migration and proliferation (29, 45). Thus, endothelial dysfunction plays a central role in the pathogenetic mechanisms underlying the development and the progression of atherosclerosis (46).

Finally, UA crystals have been reported to be able to activate the NLRP3 inflammasome, which in turns promotes the cleavage of caspase-1 and the consequent increased production of pro-inflammatory cytokines (47).

In this study, we demonstrated that UA is able to exert a direct effect on insulin signaling inducing NO synthesis, as observed in condition of insulin resistance. In fact, inhibitory effect of UA on I_R_ in endothelial cells could affect the vascular integrity ensured by the protective action of insulin. Thus, in all clinical conditions associated with an insulin resistance status, such as diabetes, obesity, hypertension, and metabolic syndrome, the presence of hyperuricemia represents an important factor capable to induce and sustain endothelial damage.

In conclusion, data obtained from this study clearly demonstrate that UA directly interfere with insulin signaling pathway, being able to inhibit the trigger of insulin signaling at receptor level through an ENPP1 recruitment. This evidence proposes a new molecular model of UA-induced insulin resistance that goes beyond the increase of oxidative stress and the promotion of inflammation. This finding attribute to UA the role of leading player in the pathogenesis of insulin resistance and endothelial dysfunction, suggesting that hyperuricemia can significantly contribute to the pathophysiological mechanisms of atherosclerosis and to the appearance of new diabetes. This has several important clinical implications because the reduction of UA levels might represent an innovative treatment goal in the prevention of both diabetes and vascular damage, two conditions that significantly worsen cardiovascular risk profile. This is particularly important, especially in view of the fact that fructose is largely used in industrial food. Importantly, since fructose is a precursor of UA (37), it would be strongly recommended to limit its use to avoid the onset of type-2 diabetes mellitus and other metabolic disorders.

## Author Contributions

ET, AC, and MH gave substantial contributions to acquisition, analysis, and interpretation of data for the work. MP, AS, FA, GS, and FP gave substantial contributions to conception, design, and interpretation of data of the work. All the authors contributed to the manuscript, provided critical revisions, approved the final version, and agreed to be accountable for all aspects of the work.

## Conflict of Interest Statement

The authors declare that the research was conducted in the absence of any commercial or financial relationships that could be construed as a potential conflict of interest.
